# Leaf nitrogen allocation to non-photosynthetic apparatus reduces mesophyll conductance under combined drought-salt stress in *Ginkgo biloba*

**DOI:** 10.3389/fpls.2025.1557412

**Published:** 2025-02-12

**Authors:** Lehao Li, Kai Zhou, Xin Yang, Xina Su, Peng Ding, Ying Zhu, Fuliang Cao, Jimei Han

**Affiliations:** ^1^ Co-Innovation Center for Sustainable Forestry in Southern China, Nanjing Forestry University, Nanjing, China; ^2^ Statistics, School of Mathematics and Statistics, Shandong University of Technology, Zibo, China

**Keywords:** combined drought-salt stress, drought stress, leaf nitrogen allocation ratio, mesophyll conductance, photosynthesis, salt stress

## Abstract

Leaf nitrogen allocation plays a crucial role in determining both photosynthetic function and structural development of plants. However, the effects of drought, salt stress, and their combination on leaf nitrogen allocation, and how these affect mesophyll conductance (*g*_m_) and photosynthesis, remain poorly understood. In this study, we first investigated variations in photosynthetic characteristics and leaf nitrogen allocation, and analyzed the relationship between *g*_m_ and leaf nitrogen allocation ratios in *Ginkgo biloba* under drought, salt and combined drought-salt stress. The results showed that all stress treatments significantly reduced the photosynthesis in *G. biloba*, with the combined drought-salt stress having the most significant inhibitory effect on the plant’s physiological characteristics. Under combined drought-salt stress, the limitation of photosynthesis due to *g*_m_ (*MC*_L_) was significantly greater than under individual drought or salt stress. In contrast, the limitation due to stomatal conductance (*S*_L_) was similar to that observed under drought but higher than under salt stress. No significant differences in biochemical limitations (*B*_L_) were found across all stress treatments. Further research suggests that the increase in *MC*_L_ under combined drought-stress treatment may be linked to a greater allocation of leaf nitrogen to non-photosynthetic apparatus (*e.g*., cell structure) and a smaller allocation to photosynthetic enzymes (i.e., ribulose-1,5-bisphosphate carboxylase/oxygenase, Rubisco). This is supported by the positive correlation between *g*_m_ and the proportion of nitrogen allocated to the carboxylation system (*P*_r_), as well as the negative correlation with the non-photosynthetic nitrogen ratio (*P*_np_). These findings help to advance our understanding of the mechanisms of photosynthesis and plant adaptability under combined drought-salt stress.

## 1 Introduction

Drought and salt stress are major abiotic factors limiting plant growth (Wang et al., 2003). With global climate change, the impact of these stresses is becoming increasingly severe in arid and semi-arid regions (Hussain et al., 2019). Plant photosynthesis is a physiological process highly sensitive to drought and salt stress (Chaves et al., 2009; Alam et al., 2021). Therefore, understanding the limiting factors and regulatory mechanisms of photosynthesis under drought and salt stress is crucial for mitigating their impact on agricultural and forestry productivity (Chaves et al., 2011).

Drought stress limits photosynthesis by restricting CO_2_ diffusion from the atmosphere to the carboxylation sites within chloroplasts (Chaves et al., 2002; Flexas et al., 2002). Stomatal closure is the first response to drought (Nadal and Flexas, 2018), typically accompanied by a reduction in stomatal conductance (*g*_s_). Meanwhile, mesophyll conductance (*g*_m_) is significantly reduced due to increased cell wall thickness (*T*_cw_) and the inhibition of aquaporins (AQPs) and carbonic anhydrase (CA) activity (Miyazawa and Terashima, 2001; Terashima et al., 2005). Similar to drought stress, salt stress reduces *g*_m_ by altering osmotic pressure and causing ion toxicity, which leads to leaf dehydration and subsequent changes in leaf anatomical structure (Tosens et al., 2012; Zait et al., 2019). Studies have shown that salt stress increases *T*_cw_ and the distance between chloroplasts and the cell wall, while reducing chloroplast density and the chloroplast surface area exposed to intercellular air space (*S*_c_/S), thereby leading to a decrease in *g*_m_ (Tosens et al., 2012; Scoffoni and Sack, 2017; Oi et al., 2020; Melo et al., 2021; Liu et al., 2021). Flexas et al. (2004) concluded that salt and drought stress primarily impact CO_2_ diffusion in the leaves by reducing *g*_s_ and *g*_m_, rather than affecting the biochemical capacity to assimilate CO_2_, at mild to moderately severe stress levels. Although the combined effects of drought and salt stress are widely recognized as a major limiting factor, research on this topic remains relatively scarce (Stavridou et al., 2019). Previous studies have shown that drought can exacerbate the negative impacts of salt stress by interfering with photosynthesis and nutrient absorption, thereby further inhibiting plant growth (Alvarez and Sánchez-Blanco, 2015; Alam et al., 2021). However, the physiological responses of leaves under combined drought-salt stress and their impact on plant photosynthetic capacity have not been thoroughly investigated.

Nitrogen is an essential nutrient for plant growth and a key factor in determining photosynthesis. Plants allocate a significant portion of leaf nitrogen to the key photosynthetic enzyme (i.e., ribulose-1,5-bisphosphate carboxylase/oxygenase - Rubisco), creating a strong link between nitrogen and photosynthetic function (Takashima et al., 2004; Damour et al., 2008; Zhong et al., 2019; Liu et al., 2022). However, leaf nitrogen is not only utilized for photosynthesis apparatus, but is also allocated to non-photosynthetic apparatus to regulate physiological traits such as *T*_cw_ (Onoda et al., 2017). Numerous studies have demonstrated that nitrogen content per area (*N*_a_) significantly influences the *g*_m_ by altering the expression of AQPs (Xiong and Flexas, 2018), the permeability of biological membranes (Flexas et al., 2006), the *T*_cw_, and the chloroplast surface area exposed to intercellular air space (*S*_c_/S) (Evans et al., 2009; Terashima et al., 2011; Tholen and Zhu, 2011). However, the effects of leaf nitrogen allocation on changes in *g*_m_ under combined drought-salt stress remain insufficiently understood.

*Ginkgo biloba*, a renowned “living fossil” and valuable relict species, is widely distributed across the globe and is known for its remarkable ecological resilience and adaptability to diverse environmental conditions (Zhao et al., 2010, 2019). Meanwhile, Wang et al. (2020) have demonstrated that *G. biloba* contains a wealth of resistance genes, which play a critical role in coping with abiotic stresses such as drought and salt. Therefore, investigating the physiological responses of *G. biloba* to stressful environments can provide new perspectives and insights for forestry breeding and the study of plant adaptation mechanisms. While research has been conducted on plant responses to environmental stresses, how leaf nitrogen allocation affects *g*_m_ and, consequently, photosynthesis remains unclear under combined drought-salt stress. This study uses *G. biloba* as experimental material and aims to 1) investigate the variations in photosynthetic traits and leaf nitrogen allocation in *G. biloba* under combined drought-salt stress; 2) explore whether leaf nitrogen allocation affects *g*_m_ under combined drought-salt stress.

## 2 Materials and methods

### 2.1 Plant material and experimental design

The experiment was conducted from June to August 2024 at the Xiashu Forestry Station of Nanjing Forestry University in Jurong City, Jiangsu Province (119°12′E, 32°07′N). This site is characterized by a subtropical monsoon climate, with abundant rainfall and ample sunlight. The average monthly environmental temperature was 28°C, and the average monthly precipitation was 139.4 mm. The 3-year-old *G. biloba* seedlings with relatively uniform plant heights were planted in 12 L flowerpots, with one plant per pot. The average height of the plants is approximately 1 meter, and the base diameter is about 1.2 centimeters. The nutrient soil was mixed with organic cultivation matrix (mixed fertilizer: perlite = 80%: 20%; organic matter content ≥ 35%; pH 5.5-7.0) and loess at a ratio of 4:1. *G. biloba* seedlings were placed in a rain-shielded greenhouse for growth, where the light level was approximately half of the natural light.

Stress treatments were applied once the sixth leaf of all plants had fully expanded. The experimental design included a control group (CK, 30% soil absolute water content - AWC + 0 mmol/L NaCl) and three treatment groups: drought treatment (D, 10% AWC + 0 mmol/L NaCl), salt treatment (S, 30% AWC + 150 mmol/L NaCl), and combined drought-salt treatment (SD, 10% AWC + 150 mmol/L NaCl). Each treatment consisted of 9-12 plants.

For the salt treatments, we began initiating the plants with NaCl solutions in early June. To avoid salt shock, NaCl concentrations of 50, 100, and 150 mmol/L were applied stepwise for three consecutive days, followed by 150 mmol/L NaCl every seven days for a total of three applications until the drought treatment began.

For the water control, all plants were initially irrigated daily to water saturation (30% AWC). To maintain a consistent AWC, all the plants were weighed and irrigated every evening. The total weight (the sum of water weight, pot weight, and dry nutrient soil weight) that needs to be maintained every day was calculated using the following equation:


(1) $$\begin{array}{c} \frac{Total\;weight\;-\;(Dry\;nutrient\;soil\;weight\;+\;Pot\;weight)\;}{Dry\;nutrient\;soil\;weight}=30\% \end{array}$$

The combined weight of the pot and dry nutrient soil was 3.5 kg, while the dry nutrient soil alone weighted 3.3 kg per pot. Note that we have omitted the weight of the plant seedlings here, as it is difficult to measure unless using destructive methods. For the drought and combined drought-salt treatments, irrigation was stopped once RWC reached the desired levels on the 18^th^ day for the drought treatment and on the 25^th^ day for the combined drought-salt treatment.

**Figure 1:**
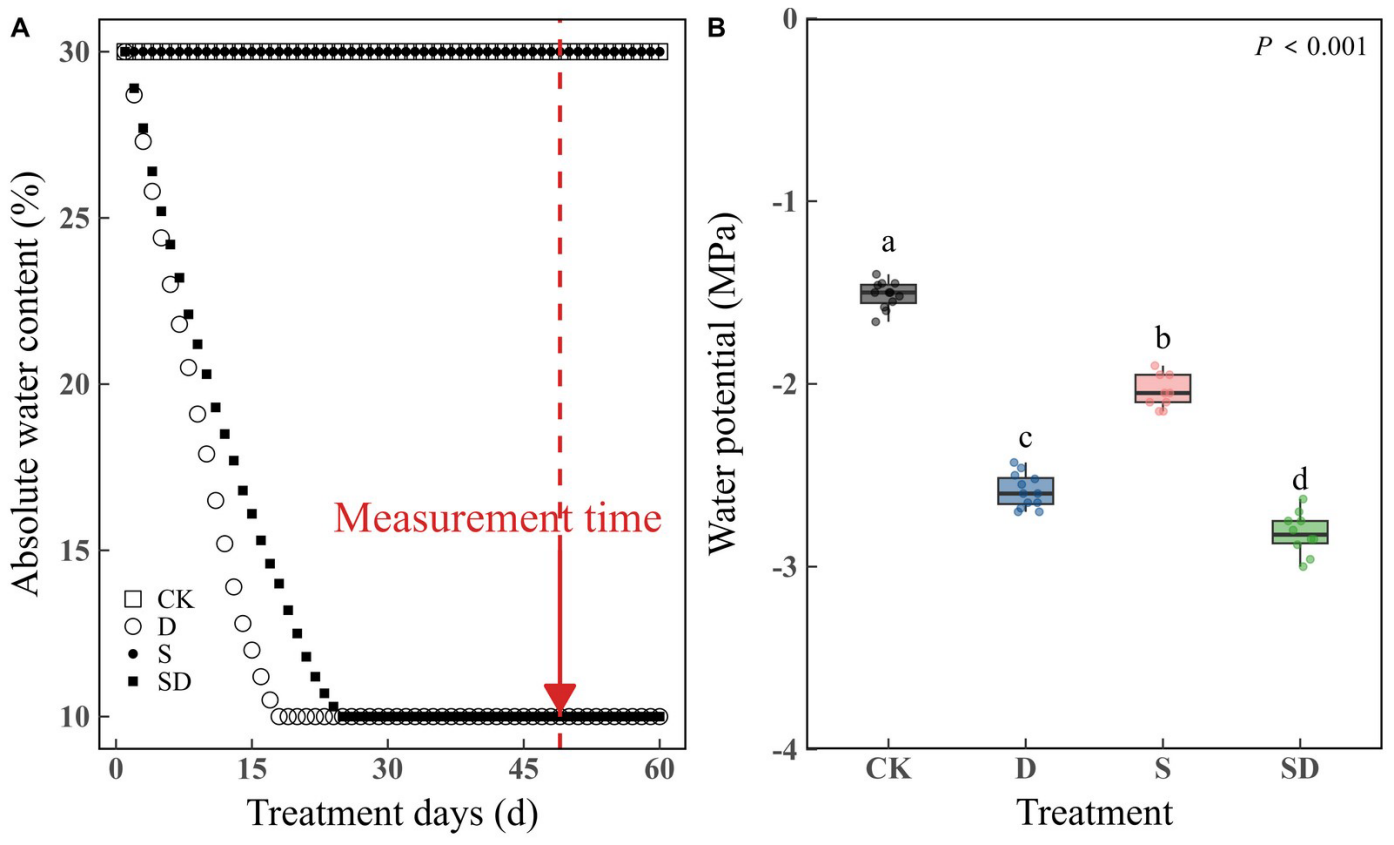
Measurement of changes in absolute soil water content and leaf water potential (*Ψ*_w_) for different stress treatments. **(A)** Differences in absolute soil water content over time for different stress treatments. **(B)** Measurement of changes in *Ψ*_w_ of different stress treatments. According to Duncan’s multiple range test, different letters indicate significant differences between stress treatments (*P* < 0.001). Each stress treatment group contained 9 to 12 samples (9≤n ≤ 12).

After 49 days of drought stress treatments, measurements were taken from the sixth mature leaf from the top of each plant (**Figure 1A**; **Supplementary Figure S2**).

### 2.2 Measurements of the concurrent gas exchange and chlorophyll fluorescence

Leaf gas exchange and chlorophyll fluorescence parameters were collected simultaneously using a Li-6800 (LI-COR, USA) equipped with a multiphase flash fluorescence leaf chamber (LI-COR, 6800-01A, 6 cm²) during sunny days. The parameters of the leaf chamber were set as follows: red/blue light ratio of 9:1, leaf temperature maintained at 30°C, relative humidity at 50%, the vapor pressure deficit (VPD) between 2.10 and 2.3 kPa, and an air flow rate set to 500 μmol s^-1^. Initially, photosynthesis was induced using a photosynthetically active radiation intensity (PPFD) of 1500 μmol m^-2^ s^-1^ and the ambient CO_2_ concentration (*C*_a_) of 400 μmol mol^-1^. After reaching a steady state in approximately 20 minutes, the CO_2_ and light response curves were measured using an automatically monitored program. The CO_2_ response curve was measured at 13 *C*_a_ values, where *C*_a_ was stepped down from 400 μmol mol^-1^ to 50 μmol mol^-1^, then increased back to 400 μmol mol^-1,^ and stepped up to 1500 μmol mol^-1^, with each step lasting 120-240 seconds. Light response curves were collected on the same leaves, with PPFD gradually decreased from 1800 μmol m^-2^ s^-1^ to 0 μmol m^-2^ s^-1^ over 12 different PPFD levels. Each step lasted 120-180 seconds, and *C*_a_ was held constant at 400 μmol mol^-1^. The gas exchange parameters (i.e., *A*_n_, intercellular CO_2_ concentration*-C*_i_, stomatal conductance to water vapor-*g*_sw_) and chlorophyll fluorescence parameters [i.e., steady-state chlorophyll fluorescence (*F*_s_) and maximum chlorophyll fluorescence (*F*_m_’)] under light conditions were obtained from the CO_2_ and light response curves. Note that stomatal conductance to CO_2_ (*g*_s_) was used in this study, and it can be calculated from *g*_sw_ (*g*_s_ = *g*_sw_/1.6). Additionally, the dark respiration rate (*R*_n_), the mimimum and maxmium chlorophyll fluorescence (*F*_o_ and *F*_m_) were measured on the same leaves under fully dark-adapted conditions. The maximum quantum yield of photosystem II (*F*_v_/*F*_m_) was calculated with *F*_o_ and *F*_m_ (*F*_v_/*F*_m =_ $\frac{F_{m}-F_{o}}{F_{m}}$ ). The measured light response curves were fit using the “photosynthesis” package in R to extract the net photosynthesis under saturating light (*A*_sat_).

### 2.3 Estimation of *g*_m_ by gas exchange and chlorophyll fluorescence

The value of *g*_m_ was estimated using the variable J approach based on Harley et al. (1992), as follows:


(2) $$\begin{array}{c} g_{m}=\frac{{\text{A}}_{\text{n}}}{{\text{C}}_{\text{i}}-\frac{{\text{Γ}}^{*}({\text{J}}_{\text{a}}\;+\;8({\text{A}}_{\text{n}}\;+\;{\text{R}}_{\text{d}}))}{{\text{J}}_{\text{a}}\;-\;4({\text{A}}_{\text{n}}\;+\;{\text{R}}_{\text{d}})}} \end{array}$$

where *R*_d_ is half of the measured dark respiration rate (*R*_n_, *R*_d_ = *R*_n_/2) (Villar et al., 1995; Piel et al., 2002; Niinemets et al., 2005), and *Γ* ^*^ represents the CO_2_ compensation point without mitochondrial respiration. According to Bernacchi et al. (2002), the value of *Γ* ^*^ at 30°C was determined and used in the calculation of *g*_m_:


(3) $$\begin{array}{c} {\text{Γ}}^{⋆}=\text{exp}(13.49-\frac{24460}{8.314\;\times \;(273.15\;+\;{\text{T}}_{\text{L}})}) \end{array}$$

where *T*_L_ is leaf temperature (°C).

The actual photochemical efficiency of PS II (*Φ*_PSII_) was calculated according to Genty et al. (1989):


(4) $$\begin{array}{c} {\text{Φ}}_{\text{PSII}}=\frac{\left({\text{F}}_{\text{m}\;}^{'}-\;{\text{F}}_{\text{s}}\right)}{{\text{F}}_{\text{m}\;}^{'}} \end{array}$$

The electron transfer rate (*J*_a_) was determined as:


(5) $$\begin{array}{c} {\text{J}}_{\text{a}}={\text{Φ}}_{\text{PSII}}\times \text{PPFD}\times \text{α}\times \text{β} \end{array}$$

where *α* represents the leaf absorptance, *β* indicates the partitioning of absorbed quanta between PS I and II. As reported by Wang et al. (2018a), no significant difference was observed in the α × β values of Oryza sativa leaves between the control group and the salt stress treatment group. Therefore, consistent values of α = 0.84 and β = 0.5 were applied to all treatments (Li et al., 2021).

### 2.4 Estimation of *g*_m_, *V*_cmax_ and *J*_max_ by the *A*_n_-*C*_c_ curve-fitting method

The *g*_m_, maximum carboxylate rate (*V*_cmax_), and maximum electron rate (*J*_max_) were estimated according to the *A*_n_-*C*_c_ curve fitting method proposed by Sharkey (2016). The variable *J* and the *A*_n_-*C*_c_ curve-fitting methods were applied to the same dataset for estimating *g*_m_. The results from the two methods were highly consistent (*R* ^2^ = 0.94, *P* < 0.001; **Supplementary Figure S1**). Therefore, only the *g*_m_ values estimated by the Harley et al. (1992) method are discussed in this study.

### 2.5 Quantitative limitations analyses of *A*_n_

According to Grassi and Magnani (2005), we analyzed the quantitative limitations of *A*_n_ by stomatal conductance (*S*_L_), mesophyll conductance (*MC*_L_) and biochemistry (*B*_L_) in *G. biloba*. The relative change of light saturation assimilation can be expressed by the parallel relative changes of *g*_s_ (*g*_sw_/1.6), *g*_m_ and *V*_cmax_, and the calculation formula is as follows:


(6) $$\begin{array}{c} \frac{\text{d}{\text{A}}_{\text{n}}}{{\text{A}}_{\text{n}}}={\text{S}}_{\text{L}}+\text{M}{\text{C}}_{\text{L}}+{\text{B}}_{\text{L}}={\text{l}}_{\text{s}}\times \frac{\text{d}{\text{g}}_{\text{s}}}{{\text{g}}_{\text{s}}}+{\text{l}}_{\text{m}}\times \frac{\text{d}{\text{g}}_{\text{m}}}{{\text{g}}_{\text{m}}}+{\text{l}}_{\text{b}}\times \frac{\text{d}{\text{V}}_{\text{cmax}}}{{\text{V}}_{\text{cmax}}} \end{array}$$

(7) $$\begin{array}{c} {\text{l}}_{\text{s}}=\frac{\frac{{\text{g}}_{\text{tot}}}{{\text{g}}_{\text{s}}}\;\times \;\frac{\partial {\text{A}}_{\text{n}}}{\partial {\text{C}}_{\text{c}}}}{{\text{g}}_{\text{tot}}\;+\;\frac{\partial {\text{A}}_{\text{n}}}{\partial {\text{C}}_{\text{c}}}} \end{array}$$

(8) $$\begin{array}{c} {\text{l}}_{\text{m}}=\frac{\frac{{\text{g}}_{\text{tot}}}{{\text{g}}_{\text{m}}}\;\times \;\frac{\partial {\text{A}}_{\text{n}}}{\partial {\text{C}}_{\text{c}}}}{{\text{g}}_{\text{tot}}\;+\;\frac{\partial {\text{A}}_{\text{n}}}{\partial {\text{C}}_{\text{c}}}} \end{array}$$

(9) $$\begin{array}{c} {\text{l}}_{\text{b}}=\frac{{\text{g}}_{\text{tot}}}{{\text{g}}_{\text{tot}}\;+\;\frac{\partial {\text{A}}_{\text{n}}}{\partial {\text{C}}_{\text{c}}}} \end{array}$$

where *g*_tot_ represents the total conductance of CO_2_ from leaf surfaces to sites of carboxylation (1/*g*_tot_ = 1/*g*_s_ + 1/*g*_m_); *l*_s_, *l*_m_ and *l*_b_ are the corresponding relative limitation (*l*_s_+*l*_m_+*l*_b_=1); ∂*A*_n_/∂*C*_c_ represents the slope of the *A*_n_-*C*_c_ curve within the range of 50-100 μmol mol ^-1^ (Tomás et al., 2013).


(10) $$\begin{array}{c} \frac{\text{d}{\text{g}}_{\text{s}}}{{\text{g}}_{\text{s}}}=\frac{\left({\text{gs}}_{\text{ref}}^{\;}\;-\;{\text{g}}_{\text{s}}\right)}{{\text{g}}_{s}^{\text{ref}}} \end{array}$$

(11) $$\begin{array}{c} \frac{\text{d}{\text{g}}_{\text{m}}}{{\text{g}}_{\text{m}}}=\frac{\left({\text{g}}_{m}^{\text{ref}}\;-\;{\text{g}}_{\text{m}}\right)}{{\text{g}}_{m}^{\text{ref}}} \end{array}$$

(12) $$\begin{array}{c} \frac{\text{d}{\text{V}}_{\text{cmax}}}{{\text{V}}_{\text{cmax}}}=\frac{\left({\text{V}}_{\text{cmax}}^{\text{ref}}\;-\;{\text{V}}_{\text{cmax}}\right)}{{\text{V}}_{\text{cmax}}^{\text{ref}}} \end{array}$$

where ${\text{g}}_{s}^{\text{ref }}$ , ${\text{g}}_{m}^{\text{ref }}$ and ${\text{V}}_{\text{cmax}}^{\text{ref }}$ are the reference values of stomatal, mesophyll conductance and maximum carboxylation rate, respectively, and were set to the maximum values observed in the control group (Flexas et al., 2009; Wang et al., 2018b).

### 2.6 Determination of leaf chlorophyll content, leaf mass per area, and nitrogen content

The leaves were placed in a 95% ethanol solution at room temperature in the dark for 48 hours until the leaves turned white. The absorbance of the extract was measured at 665 nm, 649 nm, and 470 nm using a 722 spectrophotometer. The chlorophyll content per unit area was calculated according to Arnon’s modified formula (Zhi and Xia, 2023).

A perforator with an inner diameter of 16 mm was used to obtain 10 leaf discs while avoiding the leaf veins in the leaf blade. The discs were placed in a kraft paper bag, dried at 65°C for 72 hours, and then weighed using an electronic balance. Leaf dry mass per area (LMA) was calculated by dividing leaf dry mass by leaf area, where the leaf area refers to the total area of these 10 discs, and the leaf dry mass represents the total dry mass of the same 10 discs after drying.

The leaves were removed from the petiole and dried in an oven at 65°C until constant weight, subsequently ground and passed through a 100-mesh screen. 5 mg of leaf powder was weighed for each sample and its total nitrogen content was determined using an elemental analyzer (Eurovector EA3100, Italy) following standard operating procedures.

### 2.7 Leaf water potential

Leaf water potential was measured before dawn using a Model 615 Pressure Chamber Instrument (PMS, USA). Mature leaves from the same leaf position on each plant were selected, and placed in the pressure chamber and pressurization was applied through a compressed nitrogen cylinder until the first drop of sap exudation was observed at the petiole outside the pressure chamber, and then the data were recorded.

### 2.8 Calculations of photosynthetic nitrogen use efficiency and nitrogen allocation

Leaf nitrogen allocation consists of the photosynthetic nitrogen ratio (*P*_p_) and the non-photosynthetic nitrogen ratio (*P*_np_), with *P*_p_ comprising three main components: the proportion of nitrogen allocation to light-harvesting system (*P*_L_), bioenergetics (*P*_b_) and carboxylation system (*P*_r_). *P*_L_ (nitrogen content in light-harvesting chlorophyll-protein complex), *P*_b_ (total nitrogen content of cytochrome f, ferredoxin NADP reductase, and coupling factors), and *P*_r_ (nitrogen content of Rubisco) are calculated using the estimation method proposed by Niinemets and Tenhunen (1997) as follows:


(13) $$\begin{array}{c} {\text{P}}_{\text{L}}=\frac{\text{Ch}{\text{l}}_{\text{t}}}{{\text{N}}_{\text{m}}\;\times \;{\text{C}}_{\text{B}}} \end{array}$$

(14) $$\begin{array}{c} \begin{array}{c} {\text{P}}_{\text{b}}=\frac{{\text{J}}_{\text{max}}}{8.06\;\times \;{\text{J}}_{\text{mc}}\;\times \;\text{LMA }\times \;{\text{N}}_{\text{m}}} \end{array} \end{array}$$

(15) $$\begin{array}{c} {\text{P}}_{\text{r}}=\frac{{\text{V}}_{\text{cmax}}}{6.25\;\times \;{\text{V}}_{\text{cr}}\;\times \;\text{LMA }\times \;{\text{N}}_{\text{m}}} \end{array}$$

(16) $$\begin{array}{c} {\text{P}}_{\text{np }}=100\%\;-\;{\text{P}}_{\text{p}} \end{array}$$

(17) $$\begin{array}{c} {\text{P}}_{\text{p}}={\text{P}}_{\text{L}}+{\text{P}}_{\text{b}}+{\text{P}}_{\text{r}} \end{array}$$

where *Chl*_t_ represents total leaf chlorophyll content, *N*_m_ is nitrogen content per mass. *C*_B_ refers the chlorophyll binding ratio in the photosystem, which governs the efficiency of nitrogen input into the thylakoid to participate in light harvesting and was estimated as the weighted average of chlorophyll binding to PSI, PSII and LHCII. *J*_mc_ denotes the maximum electron transfer per unit of cyt f (182.3 mol electron (mol cyt f)^-1^), *V*_cr_ is the specific activity of Rubisco (32.76 μmol CO_2_ (g Rubisco)^-1^s^-1^), 6.25 (g Rubisco (g nitrogen in Rubisco)^-1^) is the conversion factor from nitrogen content to protein content, 8.06 (μmol cyt f (g nitrogen in bioenergetics)^-1^) is the conversion factor between cyt f and nitrogen in bioenergetics.

### 2.9 Data analysis

Microsoft Excel 2019 and R software (version 4.2.2, ggplot2) were used for data collection, analysis, and drawing. One-way ANOVA was performed using the R package “agricolae” to compare mean differences between treatments, with Duncan’s test applied at a significance level of *P* < 0.05. Linear regression models for physiological traits across different treatments were fitted using the R package “ggpmisc”.

## 3 Result

### 3.1 Water status of *G. biloba* under different stress treatments

The changes in ARC with time under different treatments are shown in **Figure 1A**. The ARC was maintained at 30% in the control and salt stress treatments. Under both drought and combined drought-salt stress, we maintained the soil absolute water content at 10%. However, plants under drought stress reached the expected level more quickly than those under combined drought-salt stress. Leaf water potential (*Ψ*_w_) reflected the water status under each treatment (**Figure 1B**). As expected, *Ψ*_w_ was highest in the control, lowest under combined drought-salt stress, and higher in the salt stress treatment compared to drought stress. The change trends in *F*_v_/*F*_m_ closely align with those of *Ψ*_w_, suggesting that our water treatment effectively achieved the desired effects (**Supplementary Figure S3**; **Figure 1B**).

### 3.2 Differences in physiological traits under different treatments

**Figure 2:**
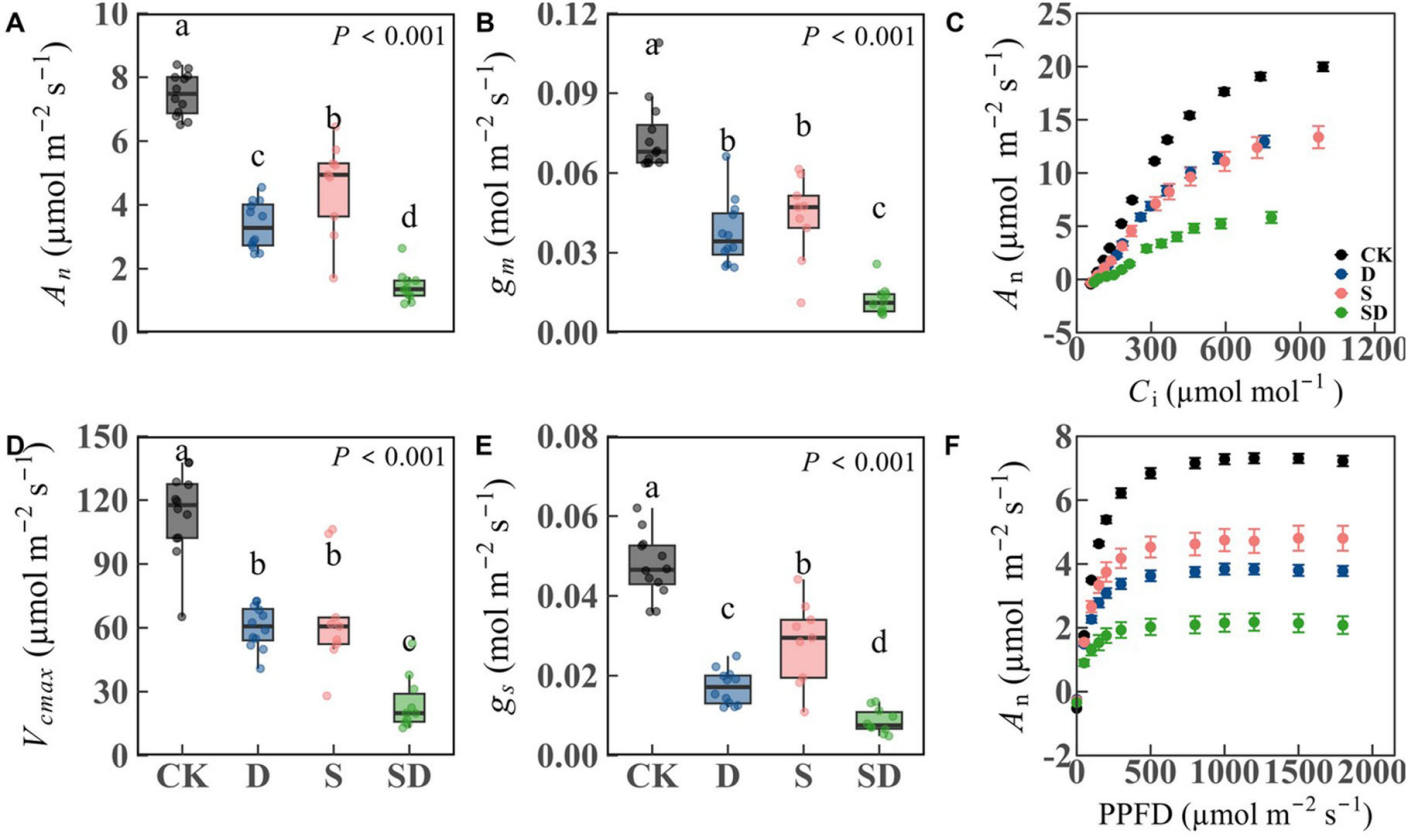
Response of photosynthetic characteristics of *G. biloba* to different stress treatments. **(A)** Net photosynthesis (*A*_n_); **(B)** Mesophyll conductance (*g*_m_); **(C)** CO_2_ response curve; **(D)** Maximum carboxylate rate (*V*_cmax_); **(E)** Stomatal conductance (*g*_s_); **(F)** Light response curve. According to Duncan’s multiple range test, different letters indicate significant differences between stress treatments (*P* < 0.001). Each stress treatment group contained 9 to 12 samples (9≤n ≤ 12).

Most physiological traits showed significant differences among treatments (**Table 1**; **Supplementary Table S1**; **Figure 2**). *A*_n_ and *g*_s_ were significantly reduced in all stress treatments compared to control, with decreases of 54.9% and 64.0% under drought stress, 39.1% and 40.5% under salt stress, and 80.5% and 81.7% under combined drought-salt stress (**Table 1**; **Figures 2A, E**). For *V*_cmax_, *J*_max_, and *g*_m_, no significant differences were observed between drought and salt stress treatments. Compared to the control, these values decreased by 47.0%, 30.1% and 49.2% under drought stress, 43.1%, 35.6% and 41.6% under salt stress, and 78.6%, 65.2% and 83.3% under combined drought-salt stress (**Table 1**; **Supplementary Table S1**; **Figures 2B, D**). Notably, since both *V*_cmax_ and *J*_max_ represent photosynthetic capacity and are linearly correlated (Walker et al., 2014), we primarily focused on the analysis of *V*_cmax_ in this study. Both drought and combined drought-salt stress significantly reduced *Chl*_t_, with no significant effect under salt stress (**Table 1**). *N*_m_ and LMA were significantly lower in all stress treatments compared to the control, but there were no differences among the stress treatments (**Table 1**). Compared to the control, *A*_sat_ decreased significantly in drought, salt and combined drought-salt stress by 47.9%, 35.1% and 68.3%, respectively (**Figure 2F**; **Table 1**).

**Table 1:** Changes in physiological traits of *G. biloba* under drought, salt stress and combined drought-salt stress treatments.

Trait	Method	Mean	SE	Min	Max	ANOVA	
*A*_sat_ (µmol m^-2^ s^-1^)	CK	8.048	0.182	6.529	8.656	a	
	D	4.197	0.188	3.348	5.556	c (-47.9%)	
	S	5.221	0.397	3.013	6.744	b (-35.1%)	***
	SD	2.554	0.301	1.390	4.279	d (-68.3%)	
*J*_max_ (µmol m^-2^ s^-1^)	CK	113.449	2.822	98.458	127.526	a	***
	D	79.288	3.744	58.375	98.347	b (-30.1%)	
	S	73.018	6.012	47.592	99.516	b (-35.6%)	
	SD	39.454	4.339	22.718	64.786	c (-65.2%)	
*Chl*_t_ (mg dm^-2^)	CK	3.951	0.176	3.126	5.076	a	***
	D	2.838	0.246	1.801	5.057	b (-28.2%)	
	S	3.611	0.148	2.872	4.162	a (-8.6%)	
	SD	2.362	0.142	1.914	3.175	b (-40.2%)	
*N*_m_ (mg g^-1^)	CK	27.453	1.156	20.190	33.260	a	**
	D	24.207	0.670	21.090	27.370	b (-11.8%)	
	S	23.904	0.991	19.540	29.120	b (-12.9%)	
	SD	21.826	0.995	18.170	28.170	b (-20.5%)	
LMA (g m^-2^)	CK	59.393	1.061	52.223	65.651	a	***
	D	49.280	0.783	43.270	52.720	b (-17.0%)	
	S	50.178	1.092	46.254	54.212	b (-15.5%)	
	SD	47.896	1.207	42.276	55.704	b (-19.4%)	

Net photosynthesis under saturated light (*A*_sat_), maximum electron transport rate (*J*_max_), total chlorophyll content (*Chl*_t_), nitrogen content per mass (*N*_m_), leaf mass per area (LMA), mean value (Mean), standard error (SE), minimum value (Min), Maximum value (Max), one-way analysis of variance (ANOVA). Each stress treatment group contained 9 to 12 samples (9≤n ≤ 12).

** and *** highly significant difference (*P* < 0.01 and *P* < 0.001) in different treatment methods. The percentage represents the change in the average value of the traits in the stress treatment group (D, S, and SD) compared with the control group (CK).

**Figure 3:**
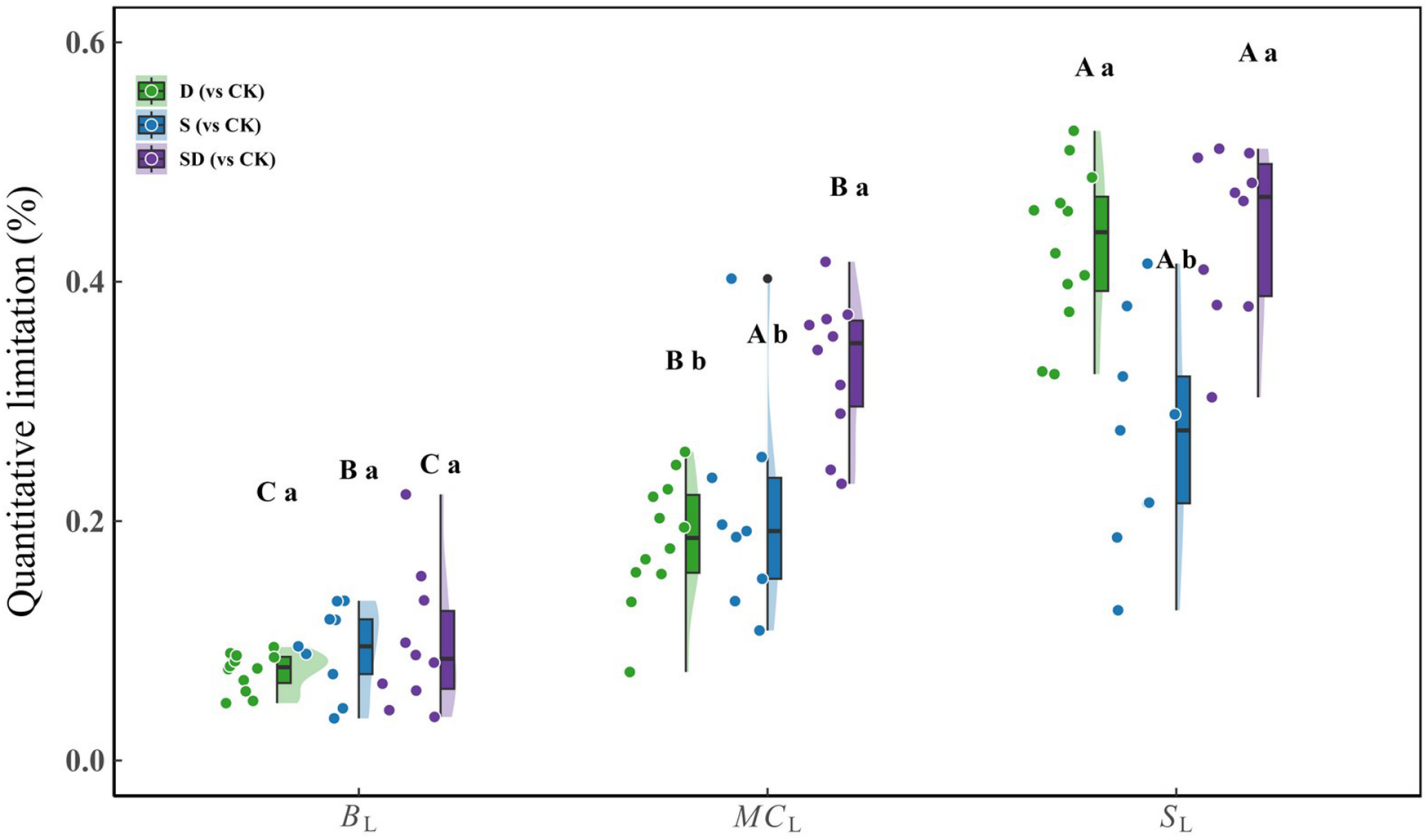
Quantitative limitation analysis of photosynthesis in *G. biloba* leaves under drought, salt, and combined drought-salt stress treatments (compared with the control group). Duncan’s test was used for multiple comparison analysis of statistical significance, with capital letters (A, B, C) representing significant differences among the three limiting factors (*S*_L_, *MC*_L_, *B*_L_) within the same treatment group; lowercase letters (a, b, c) indicate significant differences for the same limiting factor across different treatment groups (D, S, SD). Each stress treatment group contained 9 to 12 samples (9≤n ≤ 12).

Quantitative limitation analysis of photosynthesis under different stress conditions revealed significant differences in the weights of each limiting factor (**Figure 3**). There were significant differences among *B*_L_, *MC*_L_, and *S*_L_ under stress treatments, with *B*_L_ being the lowest, *MC*_L_ intermediate, and *S*_L_ the highest. Under combined drought-salt stress, *MC*_L_ was significantly greater than under individual drought or salt stress. In contrast, *S*_L_ was similar to that observed under drought but higher than under salt stress. No significant differences in *B*_L_ were found across all stress treatments.

### 3.3 Differences in leaf nitrogen allocation ratios among different stress treatments

**Figure 4:**
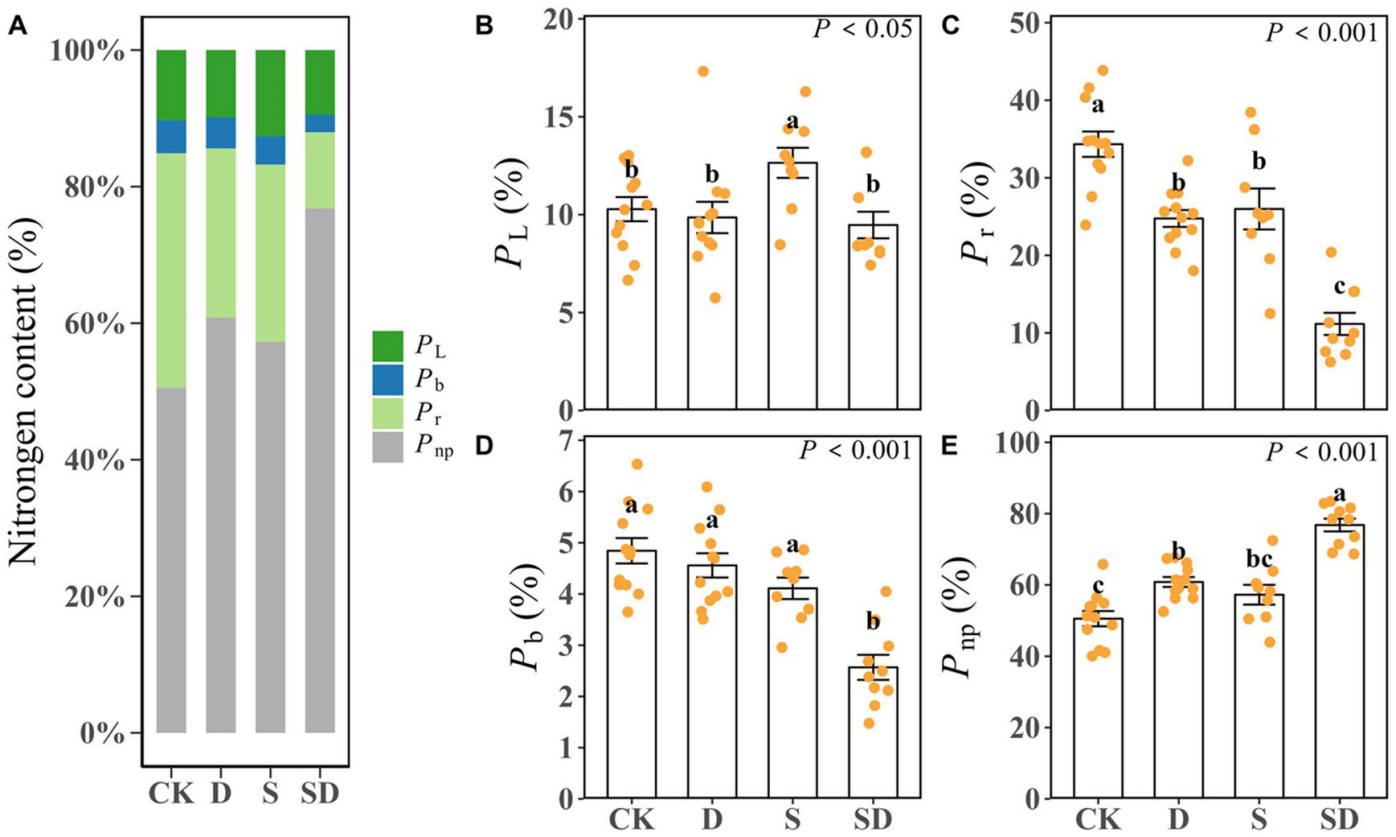
**(A)** Stacked histograms showing differences in leaf nitrogen allocation among different stress treatments. The proportion of nitrogen allocation to light-harvesting (*P*_L_), the proportion of nitrogen allocation to Rubisco (*P*_r_), the proportion of nitrogen allocation to bioenergetics (*P*_b_), the non-photosynthetic nitrogen ratio (*P*_np_). The **(B–E)** bar graph showed the differences of *P*_L_, *P*_r_, *P*_b_ and *P*_np_ among different stress treatments. Each stress treatment group contained 9 to 12 samples (9≤n ≤ 12). According to Duncan’s multiple range test, different letters indicate significant differences between stress treatments (*P* < 0.05 or *P* < 0.001).

Leaf nitrogen allocation ratios showed significant differences across the different treatments (**Table 2**; **Figure 4**). In comparison to the control, *P*_L_ was significantly increased by 23% under salt stress, yet it decreased by 4.1% and 7.9% under drought and combined drought-salt stress respectively (**Table 2**; **Figure 4B**). Drought, salt, and combined drought-salt stress led to significant reductions in *P*_b_ by 5.9%, 15.1%, and 47.0%, and there was no significant difference between drought and salt stress (**Table 2**; **Figure 4D**). *P*_r_ was significantly decreased under drought, salt and combined drought-salt stress by 27.9%, 24.3% and 67.5% respectively (**Table 2**; **Figure 4C**). In contrast, *P*_np_ was increased by 20.3%, 13.3% and 51.9% under drought, salt and combined drought-salt stress, respectively (**Table 2**; **Figure 4E**). Overall, the proportion of *P*_np_ was similar to that of *P*_p_ under all stress treatments. *P*_r_ accounted for the largest proportion of *P*_p_, followed by *P*_L_, and *P*_b_ had the smallest proportion (**Table 2**; **Figure 4**).

**Table 2:** Changes in nitrogen distribution ratio in *G. biloba* leaves under different stress treatments.

Trait	Method	Mean	SE	Min	Max	ANOVA	
*P*_L_	CK	10.280	0.616	6.658	13.026	b	*
	D	9.856	0.800	5.748	17.324	b (-4.1%)	
	S	12.647	0.768	8.463	16.280	a (23.0%)	
	SD	9.471	0.680	7.426	13.200	b (-7.9%)	
*P*_b_	CK	4.844	0.248	3.649	6.535	a	***
	D	4.558	0.237	3.507	6.089	ab (-5.9%)	
	S	4.110	0.210	2.956	4.862	b (-15.1%)	
	SD	2.568	0.245	1.474	4.050	c (-47.0%)	
*P*_r_	CK	34.331	1.633	23.914	43.841	a	***
	D	24.756	1.089	18.019	32.223	b (-27.9%)	
	S	25.980	2.643	12.493	38.436	b (-24.3%)	
	SD	11.162	1.424	6.255	20.400	b (-67.5%)	
*P*_p_	CK	49.455	2.138	34.220	59.946	a	
	D	39.170	1.387	32.308	47.477	b (-20.8%)	
	S	42.738	2.772	27.543	56.039	b (-13.6%)	***
	SD	23.201	1.792	16.577	31.323	c (-53.1%)	
*P*_np_	CK	50.545	2.138	40.054	65.780	c	***
	D	60.830	1.387	52.523	67.692	b (20.3%)	
	S	57.262	2.772	43.961	72.457	b (13.3%)	
	SD	76.799	1.792	68.677	83.423	a (51.9%)	

The proportion of nitrogen allocation to light-harvesting (*P*_L_), the proportion of nitrogen allocation to Rubisco (*P*_r_), the proportion of nitrogen allocation to bioenergetics (*P*_b_), the photosynthetic nitrogen ratio (*P*_p_), the non-photosynthetic nitrogen ratio (*P*_np_), mean value (Mean), standard error (SE), minimum value (Min), Maximum value (Max). Each stress treatment group contained 9 to 12 samples (9≤n ≤ 12).

* and *** highly significant difference (*P* < 0.05 and *P* < 0.001) in different treatment methods. The percentage represents the change in the average value of the traits in the stress treatment group (D, S, and SD) compared with the control group (CK).

### 3.4 Relationship between *g*_m_ and leaf nitrogen allocation ratio

**Figure 5:**
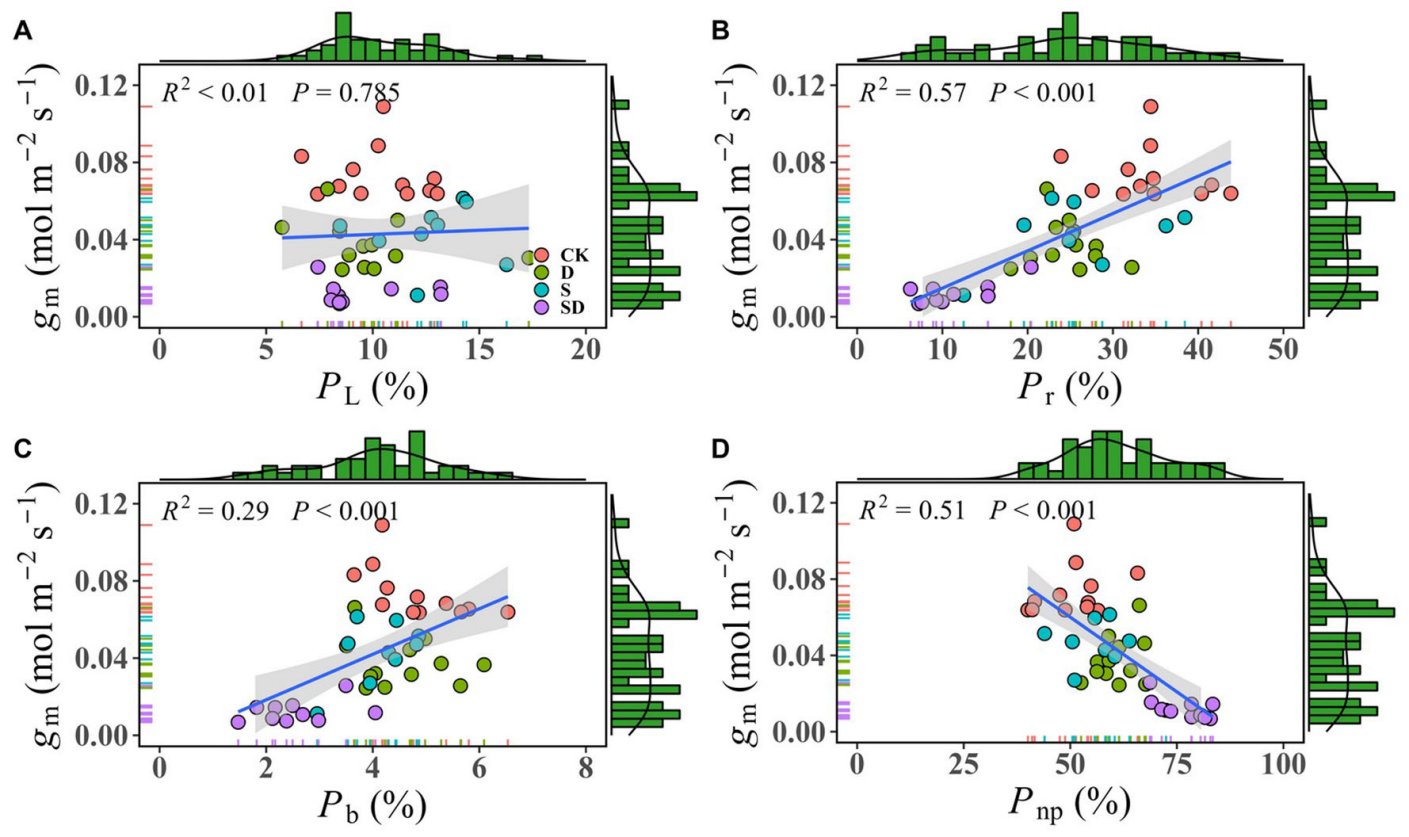
The linear correlation between *g*_m_ and leaf nitrogen allocation ratio. **(A)** The correlation between *g*_m_ and the proportion of nitrogen allocated to light-harvesting components (*P*_L_); **(B)** The correlation between *g*_m_ and the proportion of nitrogen allocated to Rubisco (*P*_r_); **(C)** The correlation between *g*_m_ and the proportion of nitrogen allocated to bioenergetics (*P*_b_); **(D)** The correlation between *g*_m_ and the non-photosynthetic nitrogen allocation ratio (*P*_np_). The marginal histograms show the frequency distribution of each variable (*g*_m_, *P*_L_, *P*_b_, *P*_L_, and *P*_np_). Each data point represents individual samples from one of four stress treatment groups: control (CK), drought (D), salt (S), and combined drought-salt (SD) stress, and each stress treatment contained 9 to 12 samples (9≤n ≤ 12).

We analyzed the correlation between *g*_m_ and leaf nitrogen distribution ratio by pooling all data together (**Figure 5**). The results showed that there was no significant relationship between *g*_m_ and with *P*_L_ (*R*² < 0.01, *P* = 0.785; **Figure 5A**). The lack of correlation between *g*_m_ and *P*_L_ likely arises because *P*_L_ is primarily influenced by *Chl*_t_, while *g*_m_ is more sensitive to leaf anatomical structure and biochemical factors. This is evidenced by the inconsistent trends in changes observed for *Chl*_t_ (**Table 1**) and *g*_m_ (**Figure 2**) under different treatments. *g*_m_ exhibited a close positive correlation with both *P*_r_ (*R*² = 0.57, *P* < 0.001; **Figure 5B**) and *P*_b_ (*R*² = 0.29, *P* < 0.001; **Figure 5C**), while showed a negative correlation with *P*_np_ (*R*² = 0.51, *P* < 0.001; **Figure 5D**).

## 4 Discussion

To investigate the role of *g*_m_ in limiting photosynthesis and its relationship with nitrogen allocation under combined drought-salt stress in *G. biloba*, we measured gas exchange and nitrogen allocation under drought, salt, and combined drought-salt treatments. The results of this study showed that *g*_m_ is considered a key factor limiting photosynthesis, especially under combined drought-salt stress. In addition, under these stress conditions, the allocation of nitrogen to photosynthetic components and non-photosynthetic apparatus plays a key regulatory role in *g*_m_, as evidenced by the significant correlations between *P*_r_ and *P*_np_ and *g*_m_.

### 4.1 Limiting factors of photosynthesis in *G. biloba* under combined drought-salt stress

Compared to the control, all three treatments resulted in significant reductions in *g*_s_, *g*_m_, and *V*_cmax_, leading to a substantial decrease in photosynthesis (**Figure 2**). This reduction can be attributed to stomatal closure, which limits CO_2_ entry, and impaired mesophyll cell structure, which increases diffusion resistance. Additionally, the decrease in *V*_cmax_ is linked to the inhibition of Rubisco activity and reduced nitrogen allocation to photosynthetic components. These findings are consistent with previous studies (Grassi and Magnani, 2005; Flexas et al., 2009; Limousin et al., 2010; Flexas, 2016; Zait et al., 2019). Further analysis revealed that the degree of decline in *g*_s_, *g*_m_, and *V*_cmax_ varied across the stress treatments. For instance, under drought conditions, *g*_s_ was significantly lower than that under salt stress, while no significant differences were observed in *g*_m_ and *V*_cmax_. This discrepancy may be attributed to the higher leaf water potential under salt stress compared to drought stress, suggesting that water potential has a more pronounced effect on stomata than on mesophyll and biochemical processes. Previous studies have also shown that stomata are the primary responders to water stress (Cornic, 2000; Chaves et al., 2002; Flexas and Medrano, 2002). When comparing single stress treatments to the combined drought-salt stress, the latter resulted in the most significant reductions in *g*_s_, *g*_m_, and *V*_cmax_, leading to the lowest photosynthetic rate. From the quantitative limitation analysis (**Figure 3**), we found that the combined drought-salt stress significantly increased *MC*_L_ relative to the individual drought or salt stress, while the *S*_L_ under combined drought-salt stress was similar to that observed under drought but higher than under salt stress. This suggests that compared to a single stress, *MC*_L_ is the dominant factor causing the significant decrease in photosynthesis under combined drought-salt stress. Under such conditions, merely regulating *g*_s_ is inadequate to alleviate the stress, prompting plants to adapt by modifying leaf structure. These structural changes can notably affect *g*_m_, leading to a considerable decrease in photosynthesis. As noted by Galmés et al. (2007), *MC*_L_ becomes a key limiting factor for photosynthesis under extreme stress conditions.

### 4.2 The shift of leaf nitrogen allocation to non-photosynthetic apparatus reduces *g*_m_ under combined drought-salt stress

Leaf nitrogen allocation is closely related to the plant photosynthetic function (Takashima et al., 2004; Evans and Clarke, 2019), with a trade-off existing between allocation to anatomical structures and photosynthetic processes (Onoda et al., 2017). In this study, we investigated whether the substantial decrease in *g*_m_ under combined drought-salt stress is associated with changes in nitrogen allocation. The results showed that, under both single drought and salt stress, the proportion of nitrogen allocation to bioenergetics (*i.e*., *P*_b_) and light-harvesting (*i.e*., *P*_L_) remained largely unchanged compared to the control, except for an increase in *P*_L_ under salt stress. However, the proportion of nitrogen allocation to Rubisco (*i.e*., *P*_r_) significantly decreased, leading to a reduced energy demand. As a result, to maintain a balance between energy production and consumption, excess energy produced during the light reaction phase might be dissipated via cyclic electron flow (Yamori and Shikanai, 2016; Pinnola and Bassi, 2018). This is supported by studies showing a significant increase in cyclic electron flow under drought and salt stress (Zivcak et al., 2013; Neto et al., 2017; He et al., 2021). Meanwhile, the reduced nitrogen allocation to Rubisco also resulted in a decreased need for CO_2_, which was reflected in lower *g*_s_ and *g*_m_. In parallel, excess nitrogen was redirected towards structural components (*e.g*., cell wall, non-photosynthetic proteins and cell membranes) (Evans and Clarke, 2019). Previous research has confirmed a close relationship between *g*_m_ and *T*_cw_ (Terashima et al., 2011; Tomás et al., 2013; Roig-Oliver et al., 2020). The increased *T*_cw_, resulting from the redistribution of nitrogen, further contributed to the reduction in *g*_m_ under drought and salt stress. Furthermore, when compared to individual stress treatments, the combined drought-salt stress led to a further significant reduction in nitrogen allocation for both energy production and Rubisco synthesis, while nitrogen allocation for non-photosynthetic functions significantly increased. This shift resulted in a further decline in the plant’s demand for CO_2_. The correlations between *P*_r_, *P*_np_, and *g*_m_ reinforce the idea that nitrogen allocation plays a crucial role in regulating *g*_m_.

Plants typically balance photosynthetic function and leaf structure construction to adapt to changes in the external environment and optimize resource utilization (Liakoura et al., 2009; Gonzalez-Paleo and Ravetta, 2018).The allocation of leaf nitrogen between photosynthetic and non-photosynthetic apparatus reflects a plant’s strategy for coordinating growth demands with environmental adaptability (Yao et al., 2018; Zhong et al., 2019). In *G. biloba*, the nitrogen allocation strategy may serve as a crucial adaptive mechanism for regulating resource distribution and enhancing survival, particularly under combined drought-salt stress. The increased allocation of nitrogen to the cell wall could play a key role in maintaining cellular function and structural integrity by enhancing the rigidity and stability of the cell wall (Feng et al., 2009), which in turn improves resistance to osmotic stress (Ridenour et al., 2008; Durand et al., 2011; Onoda et al., 2017).

### 4.3 Limitations

In this study, we adopted two methods to calculate *g*_m_ to improve reliability. However, we recognize that *g*_m_ calculations are affected by factors like photorespiration and the stability of *C*_i_, emphasizing the need for a more comprehensive model for estimating *g*_m_ (Flexas et al., 2007; Tholen et al., 2012). Additionally, we discussed the potential impact of increased *T*_cw_ on *g*_m_, although we acknowledge the absence of direct experimental data linking changes in *T*_cw_ to *g*_m_. However, previous research has suggested that the increased *T*_cw_ are closely associated with changes in *P*_np_ and reduced *P*_r_ (Feng et al., 2009). An increase in *T*_cw_ can limit *g*_m_ by restricting CO_2_ movement through the leaf tissue (Evans et al., 1994, 2009; Tosens et al., 2012; Tomás et al., 2013; Onoda et al., 2017). To gain a deeper understanding of the relationship between leaf structure and nitrogen allocation in photosynthetic tissues, further research on leaf anatomical adaptations and their effects on gas exchange is needed. Such studies would provide a more comprehensive understanding of the mechanisms underlying leaf nitrogen allocation and its impact on photosynthetic efficiency, particularly under stress conditions.

## 5 Conclusion

In summary, this study demonstrated that drought and salt stress significantly inhibited the photosynthesis in *G. biloba* by decreasing *g*_s_, *g*_m_, and *V*_cmax_, with the most pronounced negative effects observed under combined drought-salt stress. *g*_m_ was identified as a key factor limiting photosynthesis, especially under the combined drought-salt stress. Additionally, the allocation of nitrogen towards photosynthetic components (*e.g*., light-harvesting pigments, bioenergetic systems, and Rubisco) and non-photosynthetic apparatus (including cell wall, non-photosynthetic proteins and cell membranes) played a crucial regulatory role in *g*_m_ under these stress conditions, as evidenced by the significant correlation between *P*_r_ and *P*_np_ with *g*_m_.

## Data Availability

The raw data supporting the conclusions of this article will be made available by the authors, without undue reservation.
