# Supplementary material for: Leaf nitrogen allocation to non-photosynthetic apparatus reduces mesophyll conductance under combined drought-salt stress in Ginkgo biloba
Source: Front Plant Sci. 2025 Feb 12;16:1557412. doi: 10.3389/fpls.2025.1557412 (PMC11863189; doi:10.3389/fpls.2025.1557412)
Supplement: Supplementary file 1 [file DataSheet1.docx]

Supplementary Material

**Table S1** Changes in physiological traits of *G. biloba* under drought, salt stress and combined drought-salt stress treatments

| Trait | Method | Mean | SE | Min | Max | ANOVA | |
| --- | --- | --- | --- | --- | --- | --- | --- |
| *A*_n_ (µmol m⁻² s⁻¹) | CK | 7.461 | 0.194 | 6.510 | 8.393 | a |  |
|  | D | 3.364 | 0.216 | 2.467 | 4.552 | c (-54.9%) |  |
|  | S | 4.548 | 0.493 | 1.706 | 6.452 | b (-39.1%) | *** |
|  | SD | 1.454 | 0.160 | 0.903 | 2.639 | d (-80.5%) |  |
| *V*_cmax_ (µmol m⁻² s⁻¹) | CK | 113.772 | 5.881 | 65.174 | 137.659 | a | *** |
|  | D | 60.298 | 2.900 | 40.825 | 72.648 | b (-47.0%) |  |
|  | S | 64.704 | 8.445 | 28.029 | 106.239 | b (-43.1%) |  |
|  | SD | 24.402 | 3.986 | 12.963 | 52.668 | c (-78.6%) |  |
| *g*_m_ (mol m⁻² s⁻¹) | CK | 0.074 | 0.004 | 0.064 | 0.109 | a | *** |
|  | D | 0.037 | 0.004 | 0.024 | 0.066 | b (-49.2%) |  |
|  | S | 0.043 | 0.005 | 0.011 | 0.061 | b (-41.6%) |  |
|  | SD | 0.012 | 0.002 | 0.007 | 0.026 | c (-83.3%) |  |
| *g*_s_ (mol m⁻² s⁻¹) | CK | 0.047 | 0.002 | 0.036 | 0.062 | a | *** |
|  | D | 0.017 | 0.001 | 0.012 | 0.025 | c (-64.0%) |  |
|  | S | 0.028 | 0.003 | 0.011 | 0.044 | b (-40.5%) |  |
|  | SD | 0.009 | 0.001 | 0.005 | 0.013 | d (-81.7%) |  |

Net photosynthesis (*A*_n_), maximum carboxylate rate (*V*_cmax_), mesophyll conductance (*g*_m_), stomatal conductance (*g*_s_), mean value (Mean), standard error (SE), minimum value (Min), Maximum value (Max), one-way analysis of variance (ANOVA). Each stress treatment group contained 9 to 12 samples (9≤n≤12).

*** highly significant difference (*P* < 0.001) in different treatment methods. The percentage represents the change in the average value of the traits in the stress treatment group (D, S, and SD) compared with the control group (CK).


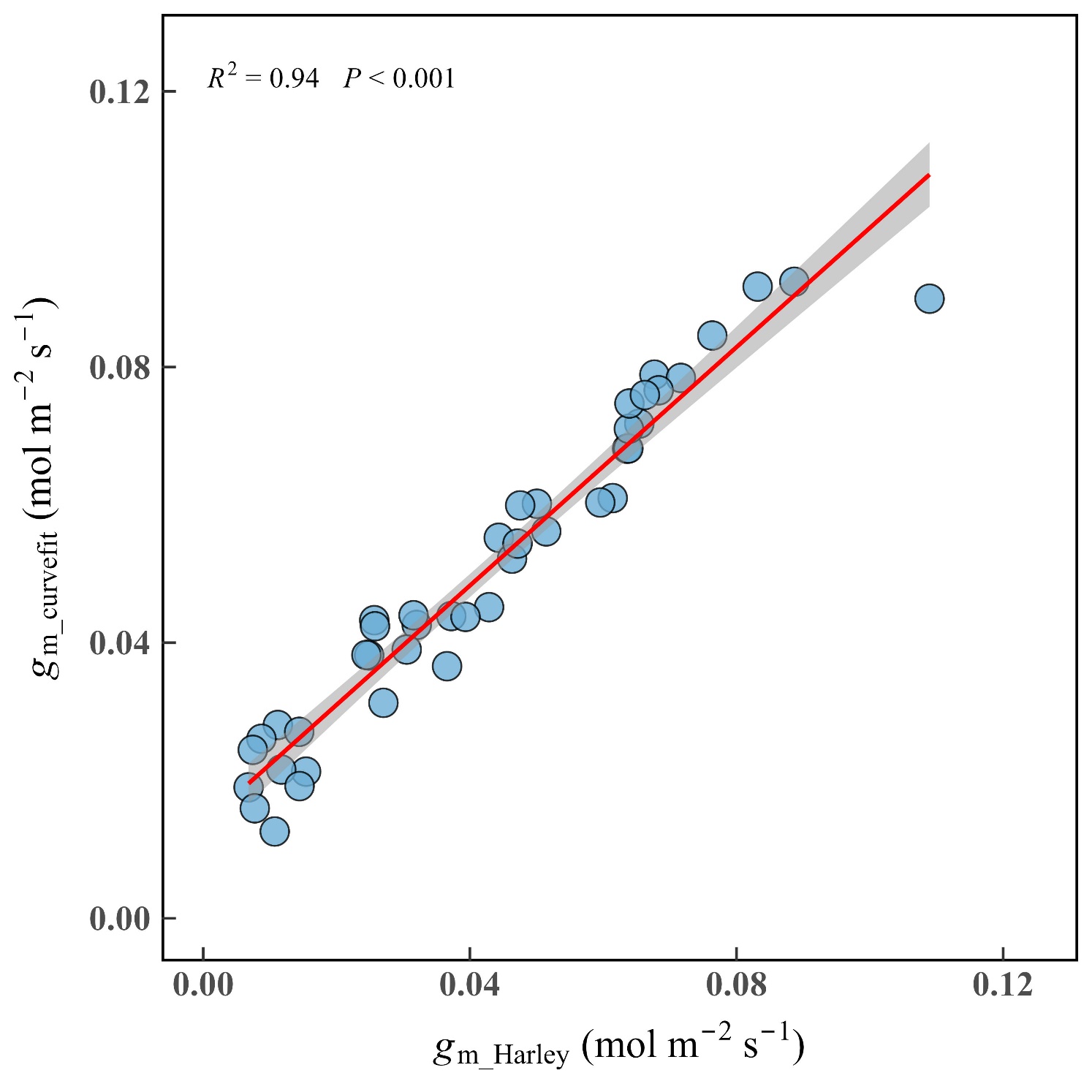


**Fig. S1.** Correlation analysis between *g*_m_curve_fitting_ and *g*_m_Harley_. The *g*_m_curve_fitting_ is fitted from the *A*_n_-*C*_c_ curve (Sharkey., 2016), and the *g*_m_Harley_ is calculated using the variable J method (Harley et al., 1992).


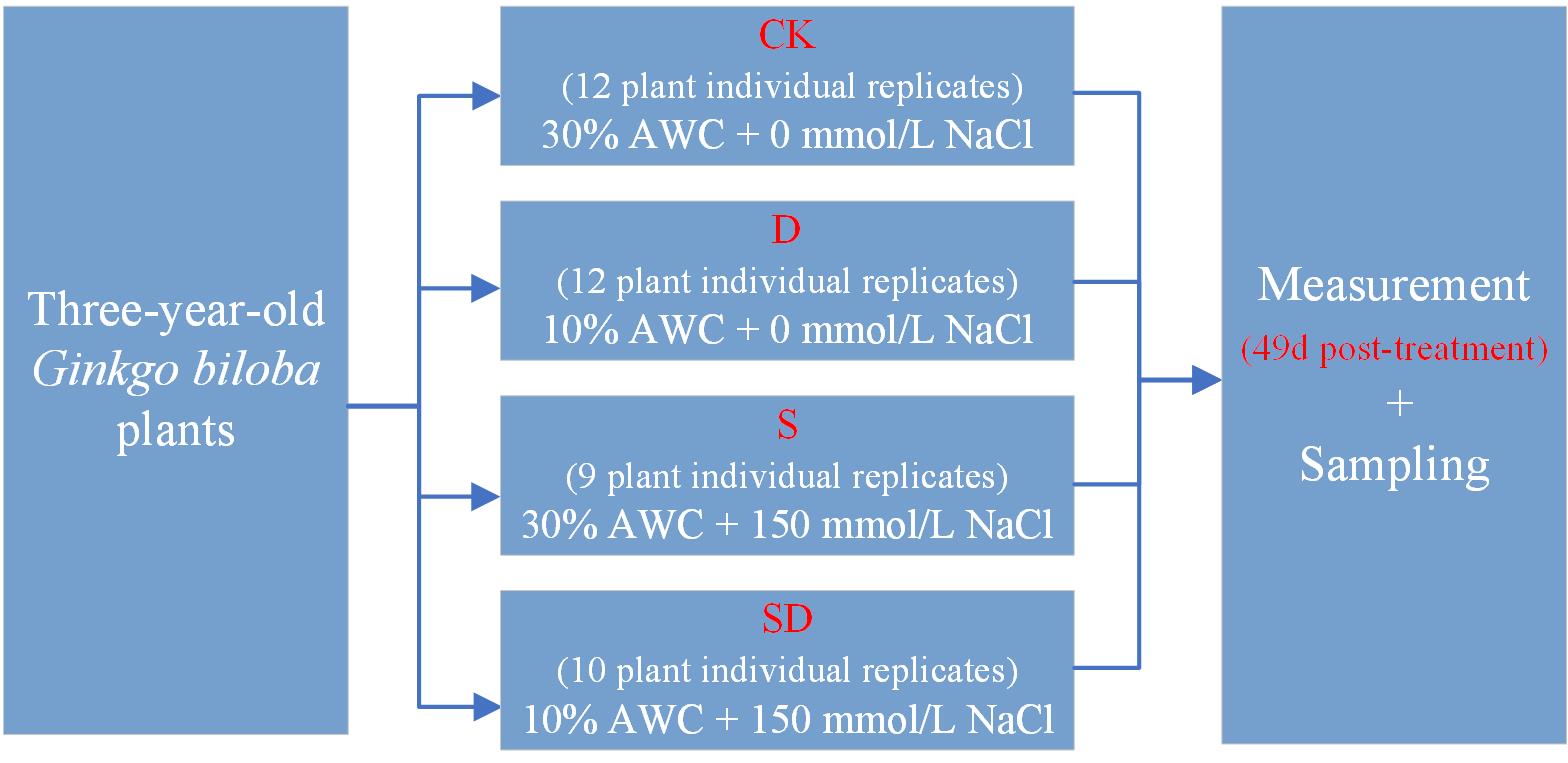


**Fig. S2.** Experimental setup flowchart.

**
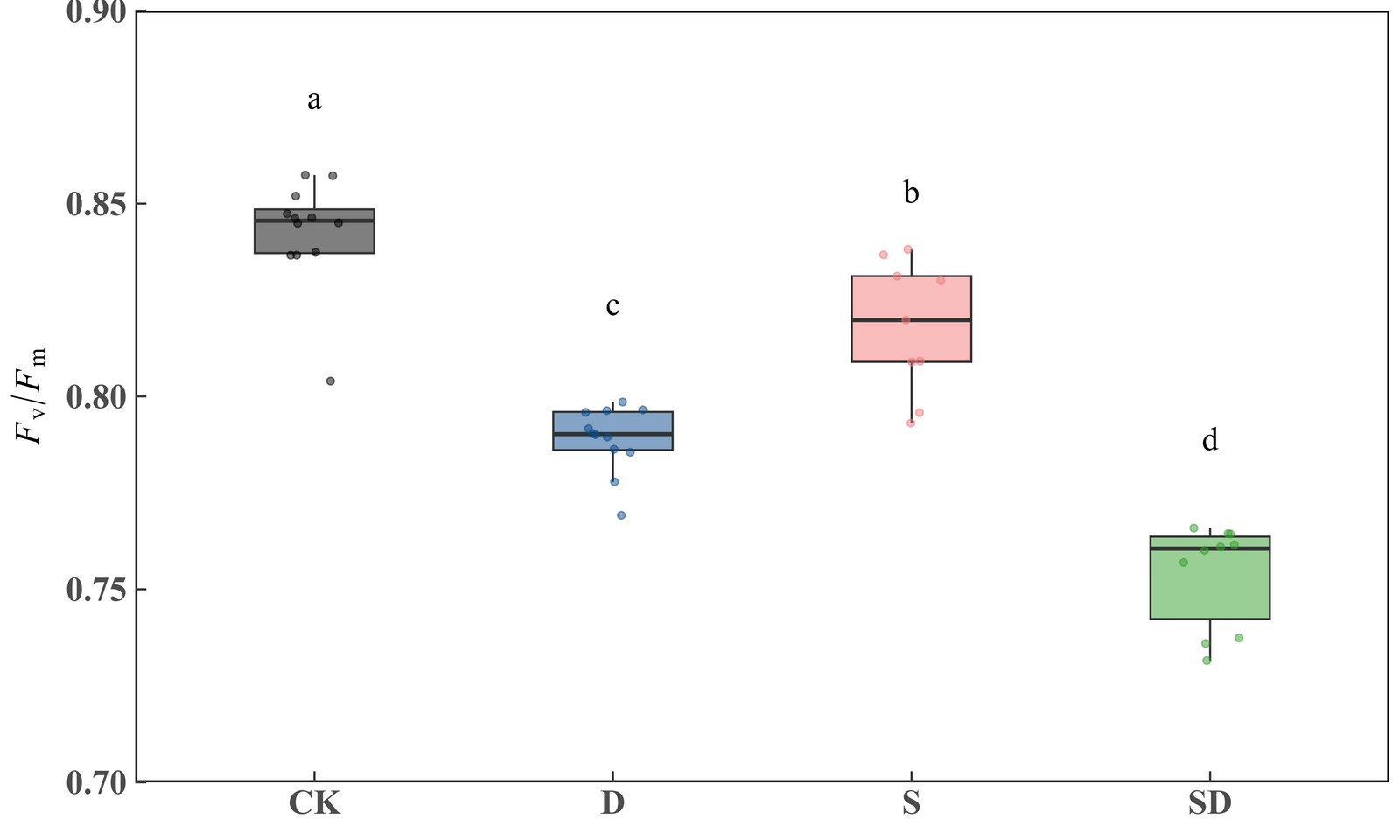
**

**Fig. S3.** Variation in the maximum quantum yield of photosystem II (*F*_v_/*F*_m_) across different stress treatments. According to Duncan's multiple range test, different letters indicate significant differences between stress treatments (*P* < 0.001). Each stress treatment group contained 9 to 12 samples (9≤n≤12).
